# Mental health conditions of young Ethiopians who use substances: a cross-sectional study in West Arsi zone

**DOI:** 10.1186/s12888-025-06550-8

**Published:** 2025-02-19

**Authors:** Jemal Ebrahim Shifa, Jon Adams, Daniel Demant

**Affiliations:** 1 School of Public Health, Faculty of Health, University of Technology Sydney, 235 Jones Street, NSW 2007 Ultimo, Australia; 2 Department of Psychiatry, Faculty of Health Sciences, Madda Walabu University, Shashemene, Ethiopia; 3 School of Public Health and Social Work, Faculty of Health, Queensland University of Technology, Queensland, Australia

**Keywords:** Young people, Substance use, Mental health, Youth mental health, Anxiety, Depression, West Arsi, Ethiopia

## Abstract

**Background:**

Mental health conditions among young Ethiopians present a pressing public health concern, posing risks to their well-being and productivity. However, there is a limited understanding of the prevalence and associated factors among young people who use substances in the West Arsi Zone, Ethiopia. This study investigated the prevalence of mental health conditions and associated factors among young people who use substances in the West Arsi Zone, Ethiopia.

**Methods:**

A community-based cross-sectional survey was conducted among 427 randomly selected young people aged 14–29 years in the West Arsi Zone of the Oromia region, Ethiopia from May 18, 2023, to September 22, 2023. Data were collected through structured interviewer-administered questionnaires. Logistic regression analysis was performed to determine the associations between the outcome and independent variables. Ethical approval was obtained from the University of Technology Sydney, Australia, and Madda Walabu University, Ethiopia.

**Results:**

A total of 424 participants were included in the analysis, giving a response rate of 99.3%. The prevalence of mental health conditions was 47% (95% CI: 40.1%, 54.2%) among substance users and 26% (95% CI: 20.3%, 32.2%) among nonusers. In the final model, among substance users, participant sex, education level, family history of substance use, and family history of mental illness remained significantly associated with mental health conditions. Among nonusers, participant sex, perceived social support, and family history of mental illness remained significant predictors of mental health conditions.

**Conclusions:**

Approximately half of the participants who used substances reported experiencing mental health conditions. This result highlights the need for appropriately focused interventions to address the growing challenges of mental health conditions and substance use among young people in Ethiopia.

**Supplementary Information:**

The online version contains supplementary material available at 10.1186/s12888-025-06550-8.

## Introduction

Young people, defined as individuals aged 10–24 years [1], constitute 24% of the global population [2]. In Africa, the proportion is greater, with 60% of the population being under 25 years old [3]. Ethiopia mirrors this trend, with 42% of its population aged 10–29 [4, 5]. Adolescence and young adulthood represent critical phases in human development, marked by significant physical, psychological, and social changes, making people particularly vulnerable to various health challenges during these periods. These challenges include substance use and mental health conditions, as well as the comorbid intersection between substance use and mental health conditions [6, 7].

Young age is a time of experimentation, exploration, curiosity, and identity search, often involving an increase in risk-taking behavior such as substance use [8, 9]. Similarly, young age is also the stage at which many mental health conditions first emerge or develop. Approximately half of all mental health conditions begin by age 14, and 75% have emerged by the mid-20s [10, 11].

The burden of mental health conditions and substance use among young people is significant and varies globally. Approximately one in seven young people is affected, representing 13% of the global burden of disease (GBD) for this age group [11]. The global prevalence of mental health conditions among adolescents is estimated to be 28% for anxiety disorders and 13% for depressive disorders [11]. In sub-Saharan Africa (SSA), including Ethiopia, research has shown high prevalence rates: 26.9% for depression, 29.8% for anxiety disorders, 21.5% for posttraumatic stress disorder (PTSD), and 20.8% for suicidal ideation [10]. In Ethiopia, mental health conditions are the leading non-communicable burden, with a prevalence of 12.3%, representing 1.3% of disability-adjusted life years (DALYs) [12]. The Ethiopian National Mental Health Strategy 2020–2025 highlights that mental health conditions in young people, with a prevalence of 12–25%, place a significant burden on the country’s health system [13].

A comorbid diagnosis of substance use and mental health conditions among young people is not uncommon, and the relationship is complex and multifaceted [14, 15]. Anxiety disorders, depression, PTSD, and suicide are the most common comorbidities [10, 16]. This comorbidity can arise from independent conditions, shared risk factors, or one influencing the other [17]. In some cases, mental health symptoms may be temporary and induced by substance intoxication or withdrawal. Substance use may develop before, concurrently with, or after the onset of a mental health condition, with an additive or cumulative interaction between the two.

The presence of mental health conditions can lead to earlier onset and more severe substance dependency and negative outcomes [15]. Similarly, substance use in those with mental health conditions can exacerbate symptoms, including suicide and suicidal ideation [14, 18]. Young people with comorbid mental health conditions and substance use often experience more negative outcomes, such as family difficulties, stigma, discrimination, homelessness, involvement in crimes, and decreased competence, support, social responsiveness, education, employment participation, and compliance with treatment [14, 17–19].

Several factors increase the risk of comorbid substance use and mental health conditions among young people in Ethiopia. Socioeconomic factors like poverty, unemployment, violence, poor education, and limited access to mental health services contribute to the cooccurrence [20, 21]. Additionally, social determinants such as family dynamics, peer pressure, and societal stigma, also play crucial roles [20, 22–24]. Further, young people may use substances to self-medicate, experience euphoria, relax, cope, fit in, or feel normal [19, 25].

Previous studies in Ethiopia have highlighted the prevalence and individual impacts of mental health conditions and substance use among young individuals [20, 21, 26–28]. While the comorbidity of these conditions has been observed in Africa, including Ethiopia, it has not been well documented, underscoring a critical need for rigorous research [11]. For example, a study revealed that khat users, one of the most commonly used substances in Ethiopia, were ten times more likely to develop depression than nonchewers were [20]. To address this gap, this study aims to determine the prevalence of mental health conditions and associated sociodemographic factors among young people who use substances in Ethiopia, thereby informing the development of integrated intervention strategies.

## Methods and materials

### Study design

A cross-sectional study was conducted from May 18, 2023, to September 22, 2023, to investigate mental health conditions among young substance users.

### Study setting

The study was conducted in the West Arsi zone of the Oromia Regional State, Ethiopia, which comprises 13 districts (woredas) and three town administrations. In 2022, the total population was approximately three million, with young people (aged 10–29 years) making up 42% or 1.2 million of this population [29]. This demographic predominance, combined with significant socio-environmental factors, makes West Arsi a particularly relevant setting for assessing the effects of substance use on mental health conditions.

The region is known for its substantial production and distribution of various psychoactive substances, including alcohol, khat, cannabis, and tobacco, which are both widely available and culturally integrated. Additionally, West Arsi experiences a high unemployment rate, reflecting broader economic challenges across Ethiopia [30], which may further heighten vulnerability. Healthcare infrastructure in West Arsi, particularly for mental health or substance-related services, is limited, restricting access to necessary support [31]. A recent study showed that nearly half of young people in West Arsi are involved in substance use [32], underscoring the critical need to understand mental health conditions and related factors among this group. Given these combined factors, assessing the mental health conditions among young substance users in this setting is important to inform targeted interventions and policy responses.

### Study population

The source population for this study included all young people residing in the West Arsi Zone. The target population consisted of individuals aged 14–29 years who had lived in the area for at least six months, were able to provide informed consent, and had reported using at least one substance in their lifetime. Young people were defined as adolescents aged 10–19 [33] and youth aged 15–29 [34]. Young individuals with severe mental disorders or cognitive impairments that prevented them from providing reliable information or informed consent were excluded from this study.

### Sample size determination and sampling procedure

This study is part of a larger research project designed to investigate substance use and mental health conditions among young people in the West Arsi Zone, Ethiopia. The sample size for the broader project was determined using a single population formula, with a 95% confidence interval, a 5% margin of error, a 10% non-response rate, and an estimated 50% prevalence of substance use among young people [35]. The full description of the sample size determination is provided elsewhere [32].

For the present research, we specifically focused on assessing the mental health conditions among young substance users. A total of 204 young individuals, aged 14 to 29 years, who reported substance use in the first phase of the project were included as the primary study sample. Mental health conditions such as depression, anxiety, PTSD, and suicidality were assessed using locally validated standard instruments (see variables and measurements section on page 9). Additionally, a comparison was made with a group of nonusers to provide context.

The study was conducted in the West Arsi zone of the Oromia Regional State, Ethiopia. A convenient and purposive sampling method was applied to select the study setting. This zone was chosen due to the researcher’s familiarity with the area, the significant production, distribution, and accessibility of various psychoactive substances, and the high unemployment rate in the region [30], which has been identified as a potential risk factor for substance use.

Among the 13 woredas in the West Arsi zone, four woredas were randomly selected, representing approximately 30% of the woredas [36]. This proportion is considered scientifically sound for ensuring representativeness while maintaining feasibility. From each selected woreda, four kebeles (the smallest administrative unit in Ethiopia) were also selected based on their accessibility and the concentration of the young population, with two kebeles drawn from urban and two from rural areas. The urban-rural stratification was included to facilitate comparisons, given documented variations in substance use between urban and rural communities in Ethiopia [37].

Following the initial step that involved the identification of the selected kebeles, and to prepare for data collection, a one-week census of households with young individuals was conducted. Each household was given a unique number to establish a sampling frame. The numbering process began at a central or predefined landmark in each kebele, such as the kebele administration office, mosque, church, school, or another prominent feature depending on the setting. From this starting point, households were numbered systematically, ensuring full coverage within the kebele boundary until the last household to be included in the study was reached.

Local young individuals, capable of writing, participated voluntarily in the process of household identification and numbering, assisted by a local law enforcement personnel assigned from the kebele administration office to ensure the safety and smooth execution of the process, particularly to the local security concerns. This was necessary due to the challenge of obtaining comprehensive information about the target population from local government sources.

The unique household identification number formed the basis for a lottery system used to randomly select households for participation. Samples were proportionally allocated among the selected kebeles to ensure representation based on the total number of young people in each kebele. Once households were identified, data collectors conducted interviews with young individuals in the selected households. In situations where more than one eligible individual was present in a household, a lottery method was applied to select one participant, ensuring randomization and avoiding clustering effects.

### Data collection process

A face-to-face interview was conducted door-to-door to administer the questionnaire. Four experts fluent in local languages conducted the data collection. Face-to-face data collection was chosen to counter the potential for information contamination as well as to ensure that participation was not inhibited by the availability of technology or literacy. Research experience in Ethiopia shows that young people tend to share information with friends and relatives when remotely administered or self-report questionnaires are used, leading to potentially biased responses and ethical concerns [38].

Following a brief introduction to the study, the participants provided both verbal and written informed consent. For participants under 18, additional written informed consent was obtained from parents and/or caregivers. The interviews lasted approximately 35–60 min.

### Measurements and variables

**The dependent variable** was mental health condition, defined as the current experience of depression, anxiety disorders, PTSD, or suicidal behavior. In this study, participants were classified as positive for mental health conditions if they responded affirmatively to at least one of these conditions, and negative otherwise. The specific mental health conditions were defined and measured as follows:

***Depression*** is measured using the Patient Health Questionnaire-9 (PHQ-9), a nine-item tool for screening for depressive disorder and assessing symptom severity [39]. The PHQ-9 scores range from 0 (absence of symptoms) to 27 (severe symptoms). Scores of 0–4 indicate no depression, 5–9 indicate mild depression, 10–14 indicate moderate depression, 15–19 indicate moderate-severe depression, and 20–27 indicate severe depression [39]. The PHQ-9 was validated in Ethiopia for use [40, 41]. Cronbach’s alpha for the scale was calculated to be 0.865, indicating a high level of internal consistency.

***Anxiety*** was measured using the Generalized Anxiety Disorder-7 (GAD-7), a seven-item tool that assesses anxiety symptoms over the past two weeks. Scores range from 0 to 21, with 0–4 indicating minimal anxiety, 5–9 indicating mild anxiety, 10–14 indicating moderate anxiety, and 15–21 indicating severe anxiety [42]. A score of 8 and above suggests an anxiety disorder [43]. The GAD-7 has been previously used in Ethiopia [44] and shows adequate internal consistency, as evidenced by a Cronbach’s alpha coefficient of 0.80 [45]. It showed high internal consistency, with a Cronbach’s alpha of 0.964, indicating well-correlated items measuring a common construct.

***PTSD***– Assessed using the Trauma Screening Questionnaire (TSQ), a ten-item tool with five “re-experiencing” and five” arousal” items. The respondents who screened positive for PTSD answered “yes” to at least six items experienced at least twice in the past week [46] and has been used in Ethiopia before [47]. The 10-item PTSD scale (ptsd1 to ptsd10) showed good internal consistency, with a Cronbach’s alpha of 0.847.

***Suicidal behavior*** - Assessed through questions on suicidal ideation, plans, and attempts. The participants were asked if they had thought about, planned, or attempted to take their own life in the past 12 months. These questions were adapted from the World Mental Health (WMH) Survey Initiative version of the WHO CIDI [48] and have been used in Ethiopian studies [49]. In the present study, we considered an affirmative response for any of the components of suicidal behavior (ideation, plan, or attempt) when counting the prevalence of suicidal behavior [49].

#### Perceived social support

Social support was measured using the 12-item Multidimensional Scale of Perceived Social Support (MSPSS) [50], previously used in Ethiopia [51] and other similar settings across Africa [52, 53]. The MSPSS has demonstrated strong internal consistency (Cronbach’s alpha values typically above 0.8) and test-retest reliability, making it a robust instrument for assessing perceived social support across different cultural and demographic contexts [54]. This study followed adaptations from comparable settings in Africa, such as Uganda [52] and Malawi [53], where slight language and context adjustments were made to preserve the instrument’s cultural relevance while maintaining its original three-factor structure [52, 53]. In this study, perceived social support levels were classified as low, moderate, and high based on the mean score [50].

#### Other demographic, social, and family factors

Other independent variables such as age, sex, education, religion, ethnicity, marital status, occupation, place of residence, children status, number of children, family size, spouse education, spouse education, family income, and source of income, family history of substance use, and family history of mental illness were assessed using a combination of author-constructed questions and adapted items from previous studies [55, 56].

### Data management and analysis

Data analysis was performed using Stata, version 18.0. Descriptive statistics were used to summarize the variables.

Simple binary logistic regression analysis was conducted for each independent variable against the dependent variable to determine the impact of each factor on mental health conditions among young people. Variables with *p* values < 0.25 in the simple binary logistic regression were included in a multiple binary logistic regression model. The effect of each independent variable on mental health conditions was assessed by controlling for potential confounders. Nonsignificant factors (*p*-value > 0.05) were removed from the final model.

Multicollinearity was assessed for the final models with a variance inflation factor (VIF) cutoff of less than five. The Hosmer–Lemeshow test confirmed the goodness of fit of the logistic regression model for predicting “any mental health condition,” yielding *p* values greater than 0.05 across all analyses, indicating a reasonable fit. Receiver operating characteristic (ROC) curve analyses revealed good discriminatory ability of the models.

### Ethical considerations

Ethics approval was obtained from the Health and Medical Research Ethics Committee at the University of Technology Sydney (ETH23-7890) and the Campus Research Ethics Review Committee of Madda Walabu University, Shashemene Campus, Ethiopia (RCSTT/34/2015). A support letter was obtained from Madda Walabu University and Woreda administration offices.

## Results

### Study sample characteristics

Table 1 shows the sociodemographic characteristics of the 424 participants included in the analysis, yielding a 99.3% response rate. The sample comprised (80%, *n* = 163) males and (20%, *n* = 41) females, with a median age of 23 years. Approximately (17%, *n* = 35) of the study samples had received basic formal education (grades 1–8), (64%, *n* = 131) were single, and (42%, *n* = 85) were employed. The majority identified as Oromo (68%, *n* = 138), (44%, *n* = 89) were Muslim, and (34%, *n* = 70) reported low perceived social support.

**Table 1:** Sociodemographic characteristics of the study sample, Ethiopia, 2024

Variable	Substance users	Nonusers
	Frequency(percentage)	Frequency(percentage)
Sex:		
Female	41(20)	112(51)
Male	163(80)	108(49)
Age in years:		
14–19	39(19)	64(29)
20–24	66(32)	58(26)
25–29	99(49)	98(45)
Participant Education:		
Not attended formal education	35(17)	26(12)
Attended basic formal education	59(29)	88(40)
Attended secondary school	54(27)	65(29)
Attended higher education	56(27)	41(19)
Marital Status:		
Single	131(64)	115(52)
Ever married	73(36)	105(48)
Participant Occupation:		
Farmer	27(13)	35(16)
Employed	85(42)	74(34)
Student	42(21)	62(28)
Unemployed	50(24)	49(22)
Place of residence:		
Rural	88(43)	105(48)
Urban	116(57)	115(52)
Ethnicity:		
Oromo	138(68)	167(76)
Amharic	24(12)	16(7)
Sidama	24(12)	22(10)
Wolaita	10(5)	8(4)
Others	8(4)	7(3)
Religion:		
Orthodox	59(29)	28(13)
Muslim	89(44)	132(60)
Protestant	48(23)	57(26)
Catholic	8(4)	3(1)
Spouse Education:		
Not attended formal education	15(21)	17(16)
Attended basic formal education	27(37)	29(28)
Attended secondary school	17(23)	37(35)
Attended higher education	14(19)	22(21)
Spouse Occupation:		
Farmer	10(14)	25(24)
Employed	36(49)	51(48)
Student	2(3)	2(2)
Unemployed	25(34)	27(26)
Children Status:		
No	144(71)	132(60)
Yes	60(29)	80(40)
Number of children:		
Less than 4 children	64(88)	87(83)
Greater than 4 children	9(12)	18(17)
Household size:		
Small family	55(27)	43(20)
Medium family	93(46)	115(52)
Large family	56(27)	62(28)
Family income per year:		
Low income	83(41)	78(35)
Medium income	82(40)	97(44)
High income	39(19)	45(21)
Source of family income:		
Agriculture	88(43)	103(47)
Trade	32(16)	25(11)
Private business	32(16)	51(23)
Salary	52(25)	41(19)
Level of social support:		
Low perceived social support	70(34)	18(8)
Moderate perceived social support	127(62)	172(78)
High perceived social support	7(4)	30(14)
Family history of substance use:		
No	91(45)	201(93)
Yes	34(17)	16(7)
Family history of mental illness:		
No	170(83)	204(93)
Yes	34(17)	16(7)
Mental health condition:		
No	108(53)	163(74)
Yes	96(47)	57(26)

### Prevalence of mental health conditions and sociodemographic distribution

The results, as illustrated in Tables 2 and 3, present the prevalence of mental health conditions and their relationships with various sociodemographic factors.

**Table 2:** The prevalence of mental health conditions among study participants, 2024

Mental health condition	Young substance users (*n* = 96/204)	Young nonusers (*n* = 57/220)
Depression		
No Depression	111(54.0)	167(75.9%)
Mild	57(28.0%)	39(17.7%)
Moderate	30(15.0%)	9(4.1%)
Moderate-severe	6(3.0%)	5(2.3%)
GAD	40(19.6%)	12(5.5%)
PTSD	14(7.0%)	15(6.8%)
Suicidal behavior	31(15.2%)	14(6.4%)
Suicidal ideation	31(15.2%)	14(6.4%)
Suicidal plan	0	2(0.9%)
Attempted suicide	2(1.0%)	1(0.5%)

**Table 3:** Factors associated with mental health conditions among young substance users and nonusers in West Arsi, Ethiopia, 2024

Variable	Mental health conditions among substance users (*N* = 204)					Mental health among conditions nonusers (220)				
	*n* = 96 (f, %)	COR 95%CI	*P* Value	AOR, 95%CI	*P* Value	*n* = 57 (f, %)	COR 95%CI	*P* Value	AOR, 95%CI	*P* Value
Sex:										
Female	26 (27.1%)	2.30(1.14, 4.67)	0.021*	3.20(1.12,9.14)	0.030*	42 (73.7%)	3.72(1.91, 7.24)	0.000*	3.19(1.51,6.74)	0.002*
Male	70 (72.9%)	Ref	Ref	Ref	Ref	15 (26.3%)	Ref	Ref	Ref	Ref
Age in years:										
14–19	12 (12.5%)	Ref	Ref	Ref	Ref	10 (17.5%)	Ref	Ref	Ref	Ref
20–24	28 (29.2%)	1.66(0.72, 3.83)	0.237	1.78(0.55,5.74)	0.335	16 (28.1%)	2.06(0.85, 4.99)	0.111	2.27(0.81,6.35)	0.120
25–29	56 (58.3%)	2.93(1.33, 6.44)	0.007*	2.70(0.75,9.70)	0.129	31 (54.4%)	2.50(1.13, 5.55)	0.024*	1.52(0.50,4.64)	0.466
Participant Education										
Not attended formal education	23 (24.0%)	2.96(1.23, 7.14)	0.016*	4.67(1.13,19.27)	0.033*	9 (15.8%)	1.28(0.48, 3.66)	0.646	1.26(0.28,5.68)	0.764
Attended basic formal education	23 (24.0%)	0.99(0.47, 2.09)	0.973	1.57(0.49,5.05)	0.452	21 (36.8%)	0.76(0.33, 1.74)	0.513	0.89(0.30,2.66)	0.838
Attended secondary school	28 (29.1%)	1.66(0.78, 3.55)	0.183	2.23(0.73,6.78)	0.158	15 (26.3%)	0.73(0.30, 1.76)	0.477	0.86(0.29,2.56)	0.782
Attended higher education	22 (22.9%)	Ref	Ref	Ref	Ref	12 (21.1%)	Ref	Ref	Ref	Ref
Marital Status:										
Single	57 (59.4%)	0.67(0.38, 1.19)	0.175	1.49(0.57,3.86)	0.413	23 (40.4%)	0.52(0.28, 0.96)	0.038*	0.61(0.24,1.54)	0.295
Ever married	39 (40.6%)	Ref	Ref	Ref	Ref	34 (59.6%)	Ref	Ref	Ref	Ref
Participant Occupation:										
Farmer	14 (14.6%)	Ref	Ref	Ref	Ref	6 (10.5%)	Ref	Ref	Ref	Ref
Employed	41 (42.7%)	0.87(0.36, 2.06)	0.743	0.88(0.19,3.78)	0.839	20 (35.1%)	1.79(0.65, 4.95)	0.262	2.18(0.59,8.12)	0.245
Student	14 (14.6%)	0.46(0.17, 1.25)	0.129	0.58(0.12,2.97)	0.517	10 (17.5%)	0.93(0.31, 2.82)	0.897	1.76(0.38,8.10)	0.468
Unemployed	27 (28.1%)	1.09(0.43, 2.78)	0.857	1.23(0.30,5.07)	0.774	21 (36.9%)	3.63(1.27, 10.31)	0.016*	2.81(0.74,10.62)	0.128
Place of residence:										
Rural	38 (39.6%)	Ref	Ref	Ref	Ref	26 (45.6%)	Ref	Ref	Ref	Ref
Urban	58 (60.4%)	1.32(0.75, 2.30)	0.334	0.92(0.40,2.11)	0.849	31 (54.4%)	1.12(0.61, 2.0.5)	0.711	0.79(0.35,1.79)	0.577
Religion:										
Orthodox	34 (35.4%)	1.91(0.98, 3.72)	0.057*	0.53(0.17,1.61)	0.259	8 (14.0%)	1.30(0.52, 3.25)	0.570	0.89(0.14,5.61)	0.901
Islam	37 (38.5%)	Ref	Ref	Ref	Ref	31 (54.4%)	Ref	Ref	Ref	Ref
Protestant	23 (24.0%)	1.29(0.64, 2.62)	0.476	1.60(0.55,4.62)	0.389	17 (29.8%)	1.38(0.69, 2.78)	0.359	1.39(0.45,4.28)	0.567
Catholic	2 (2.1%)	0.47(0.10, 2.45)	0.369	0.17(0.01,2.13)	0.170	1 (1.8%)	1.63(0.14, 18.6)	0.694	0.17(0.003,8.1)	0.368
Household size:										
Small family	23 (24.0%)	Ref	Ref	Ref	Ref	15 (26.3%)	Ref	Ref	Ref	Ref
Medium family	51 (53.1%)	1.69(0.86, 3.31)	0.127	1.19(0.45,3.15)	0.726	31 (54.4%)	0.69(0.33, 1.46)	0.330	0.74(0.29,1.93)	0.542
Large family	22 (22.9%)	0.90(0.42, 1.92)	0.786	0.95(0.31,2.94)	0.929	11 (19.3%)	0.40(0.16, 0.99)	0.049*	0.61(0.18,2.05)	0.423
Source of family income:										
Agriculture	36 (37.5%)	Ref	Ref	Ref	Ref	26 (45.6%)	Ref	Ref	Ref	Ref
Trade	21 (21.8%)	2.76(1.19, 6.41)	0.019*	3.43(0.92,12.80)	0.066	6 (10.5%)	0.94(0.34, 2.59)	0.898	0.70(0.18,2.77)	0.610
Private business	16 (16.7%)	1.44(0.64, 3.26)	0.375	1.96(0.51,7.57)	0.331	12 (21.1%)	0.91(0.42, 2.00)	0.816	0.75(0.26,2.16)	0.598
Salary	23 (24.0%)	1.46(0.57, 2.29)	0.701	1.67(0.47,5.91)	0.427	13 (22.8%)	1.38(0.62, 3.04)	0.432	0.44(0.13,1.52)	0.195
Level of perceived social support:										
Low	51 (53.1%)	6.71(1.20, 37.56)	0.030*	4.32(0.30,62.34)	0.282	12 (21.1%)	8.0(2.12, 30.15)	0.002*	6.07(1.32,27.99)	0.021*
Moderate	43 (44.8%)	1.28(0.24, 6.87)	0.774	0.57(0.04,7.86)	0.678	39 (68.4%)	1.17(0.45, 3.07)	0.745	1.25(0.42,3.76)	0.689
High	2 (2.1%)	Ref	Ref	Ref	Ref	6 (10.5%)	Ref	Ref	Ref	Ref
Family history of substance use:										
No	32 (33.3%)	Ref	Ref	Ref	Ref	51 (89.5%)	Ref	Ref	Ref	Ref
Yes	64 (66.7%)	2.41(1.36, 4.25)	0.002*	2.64(1.19,5.84)	0.017*	6 (10.4%)	1.36(0.49, 3.76)	0.556	0.72(0.19,2.72)	0.632
Family history of mental illness:										
No	65 (67.7%)	Ref	Ref	Ref	Ref	43 (75.4%)	Ref	Ref	Ref	Ref
Yes	31 (32.3%)	16.69(4.90,56.81)	0.000*	11.89(2.64,53.6)	0.001*	14 (24.6%)	26.2(5.7, 119.8)	0.000*	15.91(3.14,80.73)	0.001*
Annual family income in Ethiopian Birr:										
Low	44 (45.8%)	0.97(0.45, 2.07)	0.931	0.94(0.33,2.67)	0.910	24 (42.1%)	2.06(0.83, 5.07)	0.118	1.93(0.63,5.96)	0.251
Medium	31 (32.3%)	0.52(0.24, 1.13)	0.098	0.40(0.13,1.18)	0.096	25 (43.9%)	1.61(0.66, 3.91)	0.297	1.70(0.57,5.06)	0.337
High	21 (21.9%)	Ref	Ref	Ref	Ref	8 (14.0%)	Ref	Ref	Ref	Ref
Participants Ethnicity:										
Oromo	58 (60.4%)	Ref	Ref	Ref	ref	40 (70.2%)	Ref	Ref	Ref	Ref
Amhara	17 (17.7%)	3.35(1.30, 8.60)	0.012*	2.04(0.46,9.08)	0.351	5 (8.8%)	1.44(0.47, 4.40)	0.519	0.83(0.17,4.08)	0.823
Sidama	13 (13.6%)	1.63(0.68, 3.90)	0.272	0.92(0.22,3.80)	0.906	6 (10.5%)	1.19(0.44, 3.25)	0.733	0.74(0.20,2.76)	0.654
Wolaita	3 (3.1%)	0.59(0.15, 2.38)	0.460	0.03(0.002,0.36)	0.007*	4 (7.0%)	3.18(0.76,13.28)	0.114	1.98(0.38,10.43)	0.421
Others	5 (5.2%)	2.30(0.53, 10.00)	0.267	0.33(0.04,2.47)	0.278	2 (3.5%)	1.27(0.24, 6.80)	0.780	0.98(0.12,8.91)	0.984

*=*P* value less than 0.05

#### Mental health conditions among substance users

The overall prevalence of mental health conditions among participants who reported substance use (*n* = 204) was 47% (*n* = 96; 95% CI: 40.1, 54.2), with (3%, *n* = 6) moderate-severe depression, (20%, *n* = 40) GAD, (7%, *n* = 14) PTSD, and (15%, *n* = 31) suicidal behavior (Table 2).

A higher prevalence was observed among males (63%, *n* = 26) than in females (43%, *n* = 70). Higher rates of mental health conditions were also reported among young substance users aged 25–29 years (58%, *n* = 56), single individuals (59%, *n* = 57), and those who attended secondary school (29%, *n* = 28) than in their counterparts. Additionally, young substance users with a family history of substance use and lower perceived social support experienced mental health conditions at rates of (67%, *n* = 64) and (53%, *n* = 51), respectively, whereas (32%, *n* = 31) of participants with a family history of mental illness reported similar conditions compared to their counterparts (Table 3).

#### Mental health conditions among nonusers

The overall prevalence of mental health conditions among young participants who were not using substances (*n* = 220) was 26% (*n* = 57; 95% CI: 20.3, 32.2), with (2%, *n* = 5) moderate-severe depression, (6%, *n* = 12) GAD, (7%, *n* = 15) PTSD, and (6%, *n* = 14) suicide behavior (Table 2).

The prevalence was reported to be higher among females (37.5%, *n* = 42) than among males (14%, *n* = 15). Higher rates of mental health conditions were also reported among young nonsubstance users aged 25–29 years (54%, *n* = 31), single (59%, *n* = 57), and those who attended basic formal education (grades 1–8) (37%, *n* = 21) than in their counterparts. Additionally, young nonsubstance users with a family history of substance use and lower perceived social support experienced mental health conditions at rates of (10%, *n* = 6) and (21%, *n* = 12), respectively, whereas (25%, *n* = 14) of participants with a family history of mental illness reported mental health conditions compared with their counterparts (Table 3).

### Factors associated with mental health conditions

In the preliminary unadjusted bivariate model analysis, various covariates were examined independently for their potential associations with mental health conditions across substance users and nonuser participants. These covariates include sex, age, marital status, education, occupation, religion, place of residence, level of social support, mental health condition, family size, family income, source of income, family history of substance use, family history of mental illness, participant place of residence, and participant ethnic affiliation.

Upon conducting an adjusted model analysis, controlling for potential confounders, covariates showed a statistically significant association with the likelihood of experiencing a mental health condition across substance users and nonusers, as presented below:

***Among substance users***, sex, education level, family history of substance use, and family history of mental illness remained statistically significant predictors of mental health conditions. Compared with males, females had greater odds of having mental health conditions (AOR = 1.75, 95% CI: 1.12, 9.14; *p* = 0.007). Participants with lower education levels (grades 1–8) had approximately five times greater odds of having mental health conditions than those with higher education levels (college and above) (AOR = 4.67, 95% CI: 1.13, 19.27; *p* = 0.033). Moreover, participants with a family history of mental illness (AOR = 11.89, 95% CI: 2.64, 53.6; *p* = 0.001) and a family history of substance use (AOR = 2.64, 95% CI: 1.19, 5.84; *p* = 0.017) had substantially higher odds of having mental health conditions than did those without such a history (Table 3).

***Similarly***, ***for nonusers***, sex, perceived social support, and family history of mental illness were significantly associated with the likelihood of experiencing mental health conditions. Among nonusing participants, females had approximately three times greater odds of having mental health conditions than males did (AOR = 3.19, 95% CI: 1.51, 6.74, *p* = 0.002). Compared with those reporting high perceived social support, those reporting low perceived social support had significantly greater odds of having mental health conditions (AOR = 6.07, 95% CI: 1.32, 27.99; *p* < 0.021). Additionally, participants who had a family history of mental illness were significantly more likely to have mental health conditions than were those without such a history (AOR = 15.91, 95% CI: 3.14, 80.73; *p* = 0.001) (Table 3).

## Discussion

### Mental health conditions among young people who use substances

Nearly half (47%) of the study participants who reported substance use had experienced at least one mental health condition compared to 26% among nonusers. In countries such as Ethiopia, where access to mental healthcare services is limited [57] and stigma around mental health remains high [58], individuals may turn to substances to cope with mental health conditions. This behavior creates a cycle of temporary relief that increases dependence and, in the long term, worsens mental health symptoms [59, 60]. The social environment, including family dynamics and community norms, may also play a significant role [56]. The widespread use or acceptance of substances such as alcohol and khat in some communities in the West Arsi Zone may encourage young people to engage in substance use, increasing their risk of developing mental health conditions. Family stress, poverty, lack of parental guidance, unemployment, violence, and the social marginalization of substance users all contribute to the exacerbation of both substance use and mental health conditions [19, 58, 61, 62]. Moreover, the negative impact of substance use on behaviors and lifestyles — such as irregular sleep, poor nutrition, and risky behaviors — elevates the risk of developing further mental health conditions [9, 63, 64].

### Comparison with other studies

The prevalence of mental health conditions among substance users in our study (47%) is higher than that reported in several Ethiopian studies: 25% among Ethiopian children [62], 32% among high school students in Debre Markos [65], 31% among unemployed youth in the Gedio zone [30], and 24% among students in eastern Ethiopia [66]. International studies have shown varying rates: 44% in Nepal [67], 36% in Bangladesh [68], 17% in South Africa [69], and 24% in China [70]. However, our prevalence is lower than the 55% reported among street children in Tigray [71], 58% among students in India [72], and 59% among students in conflict-affected areas in Ethiopia [73]. This finding closely aligns with the 48% rate reported in another Ethiopian study [74] and the 50% reported in Australia [75].

### Factors contributing to variations in prevalence

The variation in the prevalence of mental health conditions among substance-using participants may be due to geographical, sociocultural, and methodological factors and other characteristics of the study participants. Methodologically, the use of different screening and diagnostic tools to measure specific mental health conditions could contribute to the variations in prevalence rates. For example, studies in Tigray, Ethiopia, used a general health questionnaire (GHQ-12), whereas studies in Kenya, Nigeria, and Ghana employed the Beck Depression Inventory (BDI) scale. In contrast, studies in Nepal and India used the Center for Epidemiological Studies Depression Scale (CES-D) to measure depression, whereas we used the PHQ-9.

The mental health conditions of the substance users in our study were significantly associated with participant sex, education level, and family history of substance use and mental illness. Compared with their male counterparts, females, those with basic education (grades 1–8), and individuals with a family history of substance use or mental illness were found to have greater mental health conditions.

The higher prevalence of mental health conditions among female substance users in our study may be related to a combination of biological, sociocultural, and environmental factors. Biologically, females have a lower tolerance for substances, leading to quicker intoxication and a greater risk of adverse effects, including mental health conditions [76–78]. In our study area, as in many parts of Ethiopia, substance use by females is often perceived as deviating from societal expectations for females [32, 79]. The stress resulting from greater societal disapproval may be associated with increased mental health conditions. An internalized stigma may disproportionately discourage females from seeking help and support, exacerbating their mental health conditions. Moreover, high levels of gender-based violence in Ethiopia, worsened by substance use, may increase the likelihood of mental health conditions among substance-using young females [80, 81]. Traditional gender roles and societal expectations may create stress and pressure for females, affecting their mental health. For example, the expectation of balancing work, caregiving, and household responsibilities can lead to increased stress levels [82, 83]. In Ethiopia, economic disparities where males are more dominant and females are dependent [84], along with lower help-seeking behavior [85] and lower literacy rates among females [86], may also contribute to the higher prevalence of mental health conditions among young substance-using females.

The higher prevalence of mental health conditions among substance-using young people in this study, who were in grades 1–8, may be related to several factors. Young people in this age group are at a critical stage of cognitive, emotional, and social development [7]. Substance use during this period can disrupt brain development, increasing vulnerability to mental health conditions. Furthermore, in rural areas such as the West Arsi Zone, limited access to mental health education and support services, including those in school areas, means that young people may not fully understand the risks of substance use or have access to necessary resources for mental health care, leading to untreated conditions [87].

This study revealed that a family history of substance use and mental illness was also associated with higher rates of mental health conditions among substance-using young people. A combination of genetic predispositions and adverse social and environmental factors may explain the high prevalence of mental health conditions in this group. A family history of substance use and mental illness may disrupt family dynamics, leading to conflict and a lack of emotional support [88, 89]. These disruptions create a stressful environment that negatively impacts the socialization of young people, which in turn negatively impacts their mental well-being, increasing their susceptibility to mental health conditions. Additionally, children with a family member with a history of substance use or mental illness are often subjected to stigma and discrimination within their communities [90]. In Ethiopia, widespread misconceptions about mental illness, such as beliefs in supernatural causes such as evil spirits or curses, contribute to the perception of affected individuals as dangerous or contagious [58, 91]. This stigma leads to social exclusion, discrimination, and increased stress, which significantly increases the risk of anxiety, depression, and other mental health conditions in these young people. This social pressure can increase stress and contribute to anxiety, depression, and other mental health conditions. Furthermore, financial burdens from medical expenses and income loss add to mental health challenges, especially when combined with a young person’s own substance use [92]. Several previous studies in Ethiopia reported similar findings, showing that a family history of substance use is associated with mental health conditions among those who use substances [93–95].

### Mental health conditions among nonsubstance user participants

The prevalence of mental health conditions among nonsubstance users was 26%, with 4% reporting moderate depression, 2% reporting moderate-severe depression, 6% reporting anxiety, 7% reporting PTSD, and 6% reporting suicide behavior. Mental health conditions among nonusers were statistically associated with sex, perceived social support, and family history of mental illness.

In this study, being female was associated with higher odds of having mental health conditions. This may be explained by high levels of gender-based violence in Ethiopia [80, 81, 96], traditional gender roles and expectations in Ethiopia [82, 83], and emotion-focused coping strategies in females that increase vulnerability to depression and anxiety [97]. In Ethiopia, the economic situation between males and females [84], lower help-seeking behavior [85], and lower literacy rates among females [86] may also contribute to the higher prevalence of mental health conditions among females.

This study revealed that a family history of mental illness was also associated with higher rates of mental health conditions among nonusers. Genetic predispositions and adverse social and environmental factors may explain this condition, which disrupts family dynamics and increases stress [88, 89]. In Ethiopia, stigma and misconceptions about mental illness may contribute to social exclusion and stress [58], further impacting mental health. Financial burdens from medical expenses and income loss add to these challenges [92].

The participants with lower perceived social support presented a greater prevalence of mental health conditions, which aligns with previous findings from Ethiopia [55, 56] and other studies [98, 99]. Enhancing social support networks in Ethiopian communities could be beneficial for addressing mental health issues.

### Summary and implications

Mental health conditions are significantly more prevalent among substance users than nonusers in our study, highlighting a strong link between substance use and mental health issues. Our data show that substance users are affected primarily by factors directly related to substance use, whereas nonusers are more influenced by social and familial support. Gender was found to be a key factor among both groups, with females being more vulnerable to mental health conditions, although the reasons may differ between groups. Family history is crucial for both groups, with substance use fueling the occurrence of mental health conditions.

The findings highlight the need for integrating mental health services into substance use programs, exploring the substance use-mental health link through further research, and providing holistic, culturally tailored care for affected individuals.

## Strengths and limitations

A major limitation of this study is its cross-sectional nature, which limits the establishment of causal relationships. Additionally, as with any survey-based research, there is a potential for self-reporting bias, where participants may underreport or overreport substance use and mental health behaviors due to social desirability or other factors that could impact the accuracy and interpretation of results. Furthermore, it is important to note that the findings of this study are specific to the West Arsi zone and might not entirely reflect the pattern of mental health conditions in other regions of Ethiopia or different sociocultural contexts.

A major strength of this study is the extensive exploration of key factors associated with mental health conditions, such as gender, age, education, social support, and family history of substance use and mental illness. Furthermore, our research methodology ensured broad participation regardless of literacy level, employing face-to-face interviews with a standard questionnaire. We selected participants to ensure diversity and achieved a high response rate (99.3%), indicative of strong engagement. Importantly, our approach minimized self-selection bias, enhancing the reliability and inclusivity of our findings.

## Conclusion and recommendations

This study sheds light on the prevalence and associated factors of mental health conditions among substance users and nonusers in young people in the West Arsi Zone, Ethiopia. Nearly half of the substance users experienced mental health conditions, whereas approximately one-fourth of the nonusers experienced mental health conditions. The key associated factors included the participant’s sex, age, education, social support, and family history of substance use and mental illness.

Addressing these issues requires a comprehensive approach. Integrating mental health services into educational and community settings, focusing on early intervention and prevention. Community outreach initiatives (e.g., awareness campaigns and stigma reduction programs involving local media and religious organizations) are essential. Culturally sensitive programs to address societal expectations and support gender-specific needs such as trauma recovery, stress management, and empowerment programs for females are needed. Enhancing social support networks and access to mental health resources is crucial for mental health prevention and promotion programs. Programs such as vocational training, educational support, and recreational activities can provide alternatives to substance use and build resilience. Family-based interventions (e.g., family counseling and parenting workshops) are also recommended because of the strong association between a family history of substance use and mental illness with mental health conditions.

Future research should further explore the causal relationships between substance use and mental health conditions to develop targeted strategies.

## Electronic supplementary material

Below is the link to the electronic supplementary material.

Supplementary Material 1

## Data Availability

The minimal dataset supporting the findings of this study is fully included in the manuscript and can be accessed from the primary author upon reasonable request if additional details are required.
