# Supplementary material for: Mental health conditions of young Ethiopians who use substances: a cross-sectional study in West Arsi zone
Source: BMC Psychiatry. 2025 Feb 19;25:151. doi: 10.1186/s12888-025-06550-8 (PMC11841329; doi:10.1186/s12888-025-06550-8)
Supplement: Supplementary file 1 — Supplementary Material 1 [file 12888_2025_6550_MOESM1_ESM.docx]

| **INTERVIEW DETAILS** | | |
| --- | --- | --- |
| Questionnaire ID | ----------- |  |
| Household address Kebele | Woreda: ------------------------  Kebele: -------------------------  House number: ---------------- |  |
| Interview contact attempt #1 | dd/mm/yyyy: [-----------------] | HH: MM [-----------] |
| Contact result #1 |  |  |
| Interview contact attempt #2 | dd/mm/yyyy: [-----------------] | HH: MM [-----------] |
| Contact result #2 |  |  |
| Interview contact attempt #3 | dd/mm/yyyy: [-----------------] | HH: MM [-----------] |
| Contact result #3 |  |  |
| Name/ID of person interviewed |  |  |
| Interviewer | Name: -------------------------- | Sign: ----------------- |
| Supervisor | Name: -------------------------- | Sign: ----------------- |
| Interview time | Start: ---------------------------- | End: ------------------ |
| Date of interview | dd/mm/yyyy: [-----------------] | HH: MM [-----------] |
| Interview conducted at…... |  |  |
| **Contact result codes: 1=Completed; 2=Not at home; 3=Postponed; 4=Refused; 5=Partly completed; 6=Incapacitated; 7=Other (specify)* | | |

| **Section I: Sociodemographic Information of the Respondents** | | | | |
| --- | --- | --- | --- | --- |
| Sex | | | [Male, Female] |  |
| Age in years | | | [----------] |  |
| Living place (where do you live, in urban or rural kebele?) | | | [urban, rural] |  |
| For how long did you live in your kebele? (year/month) | | | ------, --------- |  |
| Educational background (What is the highest level of education you have completed?) | Not attended formal education  Elementary school (grades 1-6)  Junior school (grades 7-8)  Secondary school (grades 9-12)  College and above | | |  |
| Occupation (what is your work from which you get your income or how spend your day)? | | Farming  Private organization employee  Self-employed  Volunteer  Housewife  Unemployed  Student  Government employee  Daily laborer  Other (please specify) | |  |
| What is the average annual income of your family? | |  | |  |
| What is the source of your family’s annual income? | |  | |  |
| How would you rate your family’s current income or life compared to other average families in Ethiopia? | | Very low, lower, middle, higher, very high | |  |
| Marital status (What is your current marital status? | | Single  Married  Widowed  Divorced  Separated | |  |
| Your spouse’s occupation (what is your spouse’s work from which she/he gets his/her income or how they spend their day)?)) [ask only if the respondent is married or his spouse is alive) | | Farming  Private organization employee  Self-employed  Volunteer  Housewife  Unemployed  Student  Government employee  Daily laborer  Other (please specify) -------- | |  |
| Educational background of your spouse (What is the level of education of your spouse?) | | Not attended formal education  Elementary school (grades 1-6)  Junior school (grades 7-8)  Secondary school (grades 9-12)  College and above | |  |
| Religion (what is your religion?) | |  | |  |
| Ethnicity (what is your ethnicity?) | |  | |  |
| How many people, including yourself, are there in your household? | |  | |  |
| [For female interviewees] Are you pregnant? | |  | |  |
| Do you have children (including your own children and adopted ones)? If the response is no, go to question 120 | |  | |  |
| How many children do you have? | |  | |  |
| How long does it take you to get to the nearest health center? | |  | |  |
| What is your average monthly Income in Ethiopian Birr? | |  | |  |
| Family history of mental illness? | |  | |  |
| Family history of substance use? | |  | |  |

| **Section IIa: Alcohol Use Screening Questions**   1. Have you ever drunk alcohol in your life? Yes, No 2. In the past 30 days, have you consumed any alcoholic beverages? Yes, No   **Section IIb: The Alcohol Use Disorders Identification Test: Interview Version**  Read questions as written. Record answers carefully. Begin the AUDIT by saying “Now I am going to ask you some questions about your use of alcoholic beverages during this past year.” Explain what is meant by “alcoholic beverages” by using local examples of beer, wine, Areke, etc. Code answers in terms of “standard drinks”. Place the correct answer number in the box at the right. | |
| --- | --- |
| 1. How often do you have a drink containing alcohol?   (0) Never [Skip to Qs 9-10]  (1) Monthly or less  (2) 2 to 4 times a month  (3) 2 to 3 times a week  (4) 4 or more times a week | 1. How often during the last year have you needed a first drink in the morning to get yourself going after a heavy drinking session?   (0) Never  (1) Less than monthly  (2) Monthly  (3) Weekly  (4) Daily or almost daily |
| 1. How many drinks containing alcohol do you have on a typical day when you are drinking?   (0) 1 or 2  (1) 3 or 4  (2) 5 or 6  (3) 7, 8, or 9  (4) 10 or more | 1. How often during the last year have you had a feeling of guilt or remorse after drinking?   (0) Never  (1) Less than monthly  (2) Monthly  (3) Weekly  (4) Daily or almost daily |
| 1. How often do you have six or more drinks on one occasion?   (0) Never  (1) Less than monthly  (2) Monthly  (3) Weekly  (4) Daily or almost daily,  Skip to Questions 9 and 10 if the Total Score for Questions 2 and 3 = 0 | 1. How often during the last year have you been unable to remember what happened the night before because you had been drinking?   (0) Never  (1) Less than monthly  (2) Monthly  (3) Weekly  (4) Daily or almost daily |
| 1. How often during the last year have you found that you were not able to stop drinking once you had started?   (0) Never  (1) Less than monthly  (2) Monthly  (3) Weekly  (4) Daily or almost daily | 1. Have you or someone else been injured as a result of your drinking?   (0) No  (2) Yes, but not in the last year  (4) Yes, during the last year |
| 1. How often during the last year have you failed to do what was normally expected from you because of drinking?   (0) Never  (1) Less than monthly  (2) Monthly  (3) Weekly  (4) Daily or almost daily | 1. Has a relative or friend or a doctor or another health worker been concerned about drinking or suggested you cut down?   (0) No  (2) Yes, but not in the last year  (4) Yes, during the last year |
| AUDIT Total Score= | |

| **Section IIIa: Problematic khat use screening test (PKUST-17)**   1. Have you ever chewed khat in your life? Yes, No 2. In the past 30 days, have you consumed khat? Yes, No   **Section IIIb: Problematic khat use screening test (PKUST-17)**  Now, I am going to ask you some questions about your experience using Khat in the last three months. Since Khat use is problematic for some people, it is important to know the khat use pattern of use of people. Thus, please tell me openly and correctly. All the responses will be kept confidential.  ***Note****: rarely (3 times and less); sometimes (once a week); usually (2-4 days per week); always (almost daily)* | | |
| --- | --- | --- |
| During the past three months, how often do you chew khat? | Less than once in 3 months | 0 |
|  | One to three days per month | 1 |
|  | One or two days in a week | 2 |
|  | Three or four days in a week | 3 |
|  | Daily or almost daily | 4 |
| During the past three months, when you chewed khat, on average, how much time did you spend in chewing without engaging to important task? | An hour or less | 0 |
|  | 2-3 hours | 1 |
|  | 4- 5 hours | 2 |
|  | 5-6 hours | 3 |
|  | 6 hours or more | 4 |
| In the past three months, how often do you chew khat to treat distressing experiences or depression when the khat is withdrawn? | Never | 0 |
|  | Rarely | 1 |
|  | Sometimes | 2 |
|  | Quite often | 3 |
|  | Almost always | 4 |
| In the past three months, how often you experience craving for khat? | Never | 0 |
|  | Rarely | 1 |
|  | Sometimes | 2 |
|  | Quite often | 3 |
|  | Almost always | 4 |
| In the past three months, how often do you experience distressing emotional or behavior symptoms when the khat is withdrawn? | Never |  |
|  | Rarely |  |
|  | Sometimes |  |
|  | Quite often |  |
|  | Almost always |  |
| During the past three months, how much has khat led to financial problems? | None | 0 |
|  | Mild | 1 |
|  | Moderate | 2 |
|  | Sever | 3 |
|  | Extremely | 4 |
| In the past three months, to what extent you failed to do what was expected of you at work because of chewing khat? | None | 0 |
|  | Mild | 1 |
|  | Moderate | 2 |
|  | Sever | 3 |
|  | Extremely | 4 |
| During the past three months, how often you feel depressed when you didn’t chew khat? | Never | 0 |
|  | Rarely | 1 |
|  | Sometimes | 2 |
|  | Quite often | 3 |
|  | Almost always | 4 |
| During the past three months, how often you experience irritability when you didn’t chew khat? | Never | 0 |
|  | Rarely | 1 |
|  | Sometimes | 2 |
|  | Quite often | 3 |
|  | Almost always | 4 |
| During the past three months, how often you experience vivid, unpleasant dreams when you didn’t chew khat? | Never | 0 |
|  | Rarely | 1 |
|  | Sometimes | 2 |
|  | Quite often | 3 |
|  | Almost always | 4 |
| During the past three months, how often you experience tear falling that blurred your vision when you didn’t chew khat? | Never | 0 |
|  | Rarely | 1 |
|  | Sometimes | 2 |
|  | Quite often | 3 |
|  | Almost always | 4 |
| During the past three months, how often you experience frequent yawning when you didn’t chew khat? | Never | 0 |
|  | Rarely | 1 |
|  | Sometimes | 2 |
|  | Quite often | 3 |
|  | Almost always | 4 |
| During the past three months, how often your work motivation has reduced when you didn’t chew khat? | Never | 0 |
|  | Rarely | 1 |
|  | Sometimes | 2 |
|  | Quite often | 3 |
|  | Almost always | 4 |
| During the past three months, how often you experience serious fatigue or reduced energy when you didn’t chew khat? | Never | 0 |
|  | Rarely | 1 |
|  | Sometimes | 2 |
|  | Quite often | 3 |
|  | Almost always | 4 |
| During the past three months, to what extent you have increased your amount of khat to get the desired effect? | None | 0 |
|  | Mild | 1 |
|  | Moderate | 2 |
|  | Sever | 3 |
|  | Extremely | 4 |
| During the past three months, how often did you experience restlessness when you didn’t chew khat? | Never | 0 |
|  | Rarely | 1 |
|  | Sometimes | 2 |
|  | Quite often | 3 |
|  | Almost always | 4 |
| During the past three months, how often have you experienced a marked diminished effect with continued use of the same amount of khat? | Never | 0 |
|  | Rarely | 1 |
|  | Sometimes | 2 |
|  | Quite often | 3 |
|  | Almost always | 4 |

| **Section IV: Tobacco Use Assessment Questions** | | |
| --- | --- | --- |
| Have you ever used any tobacco product in your life? | Yes | No |
| Which form (type) of tobacco product have you tried? |  |  |
| Cigarette smoking |  |  |
| Pipeful tobacco smoking |  |  |
| Chewing tobacco |  |  |
| Smoking snuff |  |  |
| Smoking shisha |  |  |
| Smoking Gaya |  |  |
| Any other types of tobacco |  |  |
| In the past 30 days, have you used any tobacco products? |  |  |
| Cigarette smoking |  |  |
| Pipeful tobacco smoking |  |  |
| Chewing tobacco |  |  |
| Smoking snuff |  |  |
| Smoking shisha |  |  |
| Smoking Gaya |  |  |
| Any other types of tobacco |  |  |

| **Section V: Cannabis Use Assessment Questions** | | |
| --- | --- | --- |
| Have you ever used cannabis/hashish/ganja in your life? | Yes | No |
| In the past 30 days, have you used cannabis/hashish/ganja? |  |  |

| **Section VI: PATIENT HEALTH QUESTIONNAIRE (PHQ-9)** | | | | | | |
| --- | --- | --- | --- | --- | --- | --- |
| Over the last 2 weeks, how often have you been bothered by any of the following problems?  (*Circle numbers in the box to indicate the answer*) | **Not at all** | **Several days** | | | **More than half the days** | **Nearly every day** |
| 1. Little interest or pleasure in doing things | 0 | 1 | | | 2 | 3 |
| 1. Feeling down, depressed, or hopeless | 0 | 1 | | | 2 | 3 |
| 1. Trouble falling or staying asleep, or sleeping too much | 0 | 1 | | | 2 | 3 |
| 1. Feeling tired or having little energy | 0 | 1 | | | 2 | 3 |
| 1. Poor appetite or overeating | 0 | 1 | | | 2 | 3 |
| 1. Feeling bad about yourself or that you are a failure or have let yourself or your family down | 0 | 1 | | | 2 | 3 |
| 1. Trouble concentrating on things, such as reading the newspaper or watching television | 0 | 1 | | | 2 | 3 |
| 1. Moving or speaking so slowly that other people could have noticed. Or the opposite being so fidgety or restless that you have been moving around a lot more than usual | 0 | 1 | | | 2 | 3 |
| 1. Thoughts that you would be better off dead, or of hurting yourself | 0 | 1 | | | 2 | 3 |
| Total PHQ-9 Score ----------- = Add Columns ----- + ----- + ----- + ----- | | | | | | |
| 1. If you checked off any problems, how difficult have these problems made it for you to do your work, take care of things at home, or get along with other people? | | |  | Not difficult at all ---------  Somewhat difficult ---------Very difficult ---------  Extremely difficult --------- | | |

| **Section VII: GENERALIZED ANXIETY DISORDERS (GAD-7)** | | | | | | |
| --- | --- | --- | --- | --- | --- | --- |
| Over the last 2 weeks, how often have you been bothered by any of the following problems?  (*Circle numbers in the box to indicate the answer*) | **Not at all** | **Several days** | | | **More than half the days** | **Nearly every day** |
| 1. Feeling nervous, anxious or on edge | 0 | 1 | | | 2 | 3 |
| 1. Not being able to stop or control worrying | 0 | 1 | | | 2 | 3 |
| 1. Worrying too much about different things | 0 | 1 | | | 2 | 3 |
| 1. Trouble relaxing | 0 | 1 | | | 2 | 3 |
| 1. Being so restless that it is hard to sit still | 0 | 1 | | | 2 | 3 |
| 1. Becoming easily annoyed or irritable | 0 | 1 | | | 2 | 3 |
| 1. Feeling afraid as if something awful might happen | 0 | 1 | | | 2 | 3 |
| ***Total GAD-7 Score*** ----------- = Add Columns ----- + ----- + ----- + ----- | | | | | | |
| 1. If you checked off any problems, how difficult have these problems made it for you to do your work, take care of things at home, or get along with other people? | | |  | Not difficult at all ---------  Somewhat difficult ---------Very difficult ---------  Extremely difficult --------- | | |

| **Section VIII: Trauma Screening Questionnaire (TSQ)** | | |
| --- | --- | --- |
| Now, I am going to ask you some questions about reactions that sometimes occur after a traumatic event. The questionnaire is designed to assess personal reactions to any traumatic event that might happen to you. Please respond as (Yes/No) whether or not you have experienced any of the following at least twice in the past week. | Yes | No |
| 1. Upsetting thoughts or memories about the event that have come into your mind against your will |  |  |
| 1. Upsetting dreams about the event |  |  |
| 1. Acting or feeling as though the event were happening again |  |  |
| 1. Feeling upset by reminders of the event |  |  |
| 1. Bodily reactions (such as fast heartbeat, stomach churning, sweatiness, dizziness) when reminded of the event |  |  |
| 1. Difficulty falling or staying asleep |  |  |
| 1. Irritability or outbursts of anger |  |  |
| 1. Difficulty concentrating |  |  |
| 1. Heightened awareness of potential dangers to yourself and others |  |  |
| 1. Being jumpy or being startled at something unexpected |  |  |

| **Section IX: Suicidal Behavior Assessment Questions** |  |  |
| --- | --- | --- |
| Please mark with a cross symbol in the box that respondents answer best fits what he/she has felt or experienced in the last year | Yes | No |
| 1. Have you felt that life is not worth living? |  |  |
| 1. Have you wished you were dead? For example, going to sleep and wishing you would not get up. |  |  |
| 1. Have you thought about taking your life even if you weren’t really going to? |  |  |
| 1. Have you reached the point where you considered actually taking your own life or you made plans about how you would do it? |  |  |
| 1. Have you tried to take your own life? |  |  |

| **Section X: WHO Quality of Life Scale-Brief (WHOQOL-BREF)** | | | | | | | | | | | | | | | | | |
| --- | --- | --- | --- | --- | --- | --- | --- | --- | --- | --- | --- | --- | --- | --- | --- | --- | --- |
| Now, I am going to ask you some questions that assesses your feelings. Each question has options with scale ranging from 1-5 and choose the alternative that best fits to your feeling. | | | | | | | | | | | | | | | | | |
| 1. How would you rate your quality of life? | | | Very poor (1) | | | | Poor (2) | | Neither poor nor good (3) | | | | | Good (4) | | Very Good (5) | |
| 1. How satisfied are you with your health? | Very dissatisfied (1) | | | | | Dissatisfied (2) | | | | Neither satisfied nor dissatisfied (3) | | | | Satisfied (4) | | Very satisfied (5) | |
| The following questions ask about how much you have experienced certain things in the last two weeks. ***[Not at all = 1, A little = 2, A moderate amount= 3, Very much= 4, An extreme amount= 5]*** | | | | | | | | | | | | | | | | | |
| 1. To what extent do you feel that physical pain prevents you from doing what you need to do? | | | | | 1 | | | 2 | | | | 3 | | 4 | | 5 | |
| 1. How much do you need any medical treatment to function in your life? | | | | | 1 | | | 2 | | | | 3 | | 4 | | 5 | |
| 1. How much do you enjoy life? | | | | | 1 | | | 2 | | | | 3 | | 4 | | 5 | |
| 1. To what extent do you feel your life to be meaningful? | | | | | 1 | | | 2 | | | | 3 | | 4 | | 5 | |
| 1. How well are you able to concentrate? | | | | | 1 | | | 2 | | | | 3 | | 4 | | 5 | |
| 1. How safe do you feel in your daily life? | | | | | Not at all | | | Slightly | | | | A moderate amount | | Very much | | Extremely | |
| 1. How healthy is your physical environment? | | | | | Not at all | | | Slightly | | | | A moderate amount | | Very much | | Extremely | |
| The following questions ask about how completely you experience or were able to do certain things in the last two weeks.  ***[Not at all=1, A little=2, Moderately=3, Mostly=4, Completely=5]*** | | | | | | | | | | | | | | | | | |
| 1. Do you have enough energy for everyday life? | | | 1 | | | | 2 | | | | | 3 | | 4 | | 5 | |
| 1. Are you able to accept your bodily appearance? | | | 1 | | | | 2 | | | | | 3 | | 4 | | 5 | |
| 1. Have you enough money to meet your needs? | | | 1 | | | | 2 | | | | | 3 | | 4 | | 5 | |
| 1. How available to you is the information that you need in your day-to-day life? | | | 1 | | | | 2 | | | | | 3 | | 4 | | 5 | |
| 1. To what extent do you have the opportunity for leisure activities? | | | 1 | | | | 2 | | | | | 3 | | 4 | | 5 | |
| 1. How well are you able to get around? | | | Very poor (1) | | | | Poor (2) | | | | | Neither poor nor well (3) | | Well (4) | | Very well (5) | |
| 1. How satisfied are you with your sleep? | | | Very dissatisfied (1) | | | | Dissatisfied (2) | | | | | Neither satisfied nor dissatisfied (3) | | Satisfied (4) | | Very satisfied (5) | |
| 1. How satisfied are you with your ability to perform your daily living activities? | | | Very dissatisfied (1) | | | | Dissatisfied (2) | | | | | Neither satisfied nor dissatisfied (3) | | Satisfied (4) | | Very satisfied (5) | |
| 1. How satisfied are you with your capacity for work? | | | Very dissatisfied (1) | | | | Dissatisfied (2) | | | | | Neither satisfied nor dissatisfied (3) | | Satisfied (4) | | Very satisfied (5) | |
| 1. How satisfied are you with yourself? | | | Very dissatisfied (1) | | | | Dissatisfied (2) | | | | | Neither satisfied nor dissatisfied (3) | | Satisfied (4) | | Very satisfied (5) | |
| 1. How satisfied are you with your personal relationships? | | | Very dissatisfied (1) | | | | Dissatisfied (2) | | | | | Neither satisfied nor dissatisfied (3) | | Satisfied (4) | | Very satisfied (5) | |
| 1. How satisfied are you with your sex life? | | | Very dissatisfied (1) | | | | Dissatisfied (2) | | | | | Neither satisfied nor dissatisfied (3) | | Satisfied (4) | | Very satisfied (5) | |
| 1. How satisfied are you with the support you get from your friends? | | | Very dissatisfied (1) | | | | Dissatisfied (2) | | | | | Neither satisfied nor dissatisfied (3) | | Satisfied (4) | | Very satisfied (5) | |
| 1. How satisfied are you with the conditions of your living place? | | | Very dissatisfied (1) | | | | Dissatisfied (2) | | | | | Neither satisfied nor dissatisfied (3) | | Satisfied (4) | | Very satisfied (5) | |
| 1. How satisfied are you with your access to health services? | | | Very dissatisfied (1) | | | | Dissatisfied (2) | | | | | Neither satisfied nor dissatisfied (3) | | Satisfied (4) | | Very satisfied (5) | |
| 1. How satisfied are you with your mode of transportation? | | Very dissatisfied (1) | | | | | Dissatisfied (2) | | | | | Neither satisfied nor dissatisfied (3) | | | Satisfied (4) | | Very satisfied (5) |
| The following question refers to how often you have felt or experienced certain things in the last two weeks. | | | | | | | | | | | | | | | | | |
| 1. How often do you have negative feelings, such as blue mood, despair, anxiety, & depression? | | | | Never (1) | | | Seldom (2) | | | | Quite often (3) | | Very often (4) | | | Always (5) | |

**Section XI: Multi-dimensional Scale of Perceived Social Support (MSPSS) items**

1. Is there a special person who is around when you are in need? [strongly disagree (0), disagree (1), neutral (2), agree (3), strongly agree (4)]
2. Is there a special person with whom you can share your joys and sorrows? [strongly disagree (0), disagree (1), neutral (2), agree (3), strongly agree (4)]
3. Does your family really try to help you? [strongly disagree (0), disagree (1), neutral (2), agree (3), strongly agree (4)]
4. Do you get the emotional help and support you need from your family? [strongly disagree (0), disagree (1), neutral (2), agree (3), strongly agree (4)]
5. Do you have a special person who is a real source of comfort to you? [strongly disagree (0), disagree (1), neutral (2), agree (3), strongly agree (4)]
6. Do your friends really try to help you? [strongly disagree (0), disagree (1), neutral (2), agree (3), strongly agree (4)]
7. Can you count on your friends when things go wrong? [strongly disagree (0), disagree (1), neutral (2), agree (3), strongly agree (4)]
8. Can you talk about your problems with your family? [strongly disagree (0), disagree (1), neutral (2), agree (3), strongly agree (4)]
9. Do you have friends with whom you can share your joys and sorrows? [strongly disagree (0), disagree (1), neutral (2), agree (3), strongly agree (4)]
10. Is there a special person in your life who cares about your feelings? [strongly disagree (0), disagree (1), neutral (2), agree (3), strongly agree (4)]
11. Is your family willing to help you make decisions? [strongly disagree (0), disagree (1), neutral (2), agree (3), strongly agree (4)]
12. Can you talk about your problems with your friends? [strongly disagree (0), disagree (1), neutral (2), agree (3), strongly agree (4)]

**Section XII: Help-Seeking Measures**

1. **General Help-Seeking Questionnaire**: The following list of questions assesses your ***future help-seeking intention*** for your substance use and mental health conditions

| Below is a list of sources or people whom you might seek help or advice from if you were experiencing a personal or emotional problem. Please circle the number that shows how likely is it that you would seek help from each of these people for ***your substance use or mental health conditions*** during the **next 4 weeks**. | | | | | | | | |
| --- | --- | --- | --- | --- | --- | --- | --- | --- |
| **S.#** | **Sources of help** | ***Extremely Unlikely Extremely Likely*** | | | | | | |
|  | Intimate partner (e.g. boyfriend/girlfriend/ husband/wife) | 1 | 2 | 3 | 4 | 5 | 6 | 7 |
|  | Friend (not related to you) |  |  |  |  |  |  |  |
|  | Parent |  |  |  |  |  |  |  |
|  | Other relative / family member |  |  |  |  |  |  |  |
|  | Mental health professional (e.g., school counsellor, psychologist, psychiatrist) |  |  |  |  |  |  |  |
|  | Religious leaders (e.g. Du’a, Tselot) |  |  |  |  |  |  |  |
|  | Religious places (ex. holy water) |  |  |  |  |  |  |  |
|  | Traditional healer |  |  |  |  |  |  |  |
|  | General practitioners |  |  |  |  |  |  |  |
|  | Nurses or other health professionals |  |  |  |  |  |  |  |
|  | Teacher (year advisor, classroom teacher) |  |  |  |  |  |  |  |
|  | Someone else not listed above (please describe who this was) ________________________ |  |  |  |  |  |  |  |
|  | I would not seek help from anyone |  |  |  |  |  |  |  |

1. **General Help-Seeking Questionnaire**: The following list of questions assesses your past help-seeking experience for your substance use and mental health conditions
2. Have you ever seen a mental health professional (e.g., counsellor, psychologist, psychiatrist) to get help for your substance use or mental health conditions? (Circle one)

**Yes** **No**

If you circled “no” in question 2a, you finished this section. *If you circled “yes” please complete 2b, 2c, and 2d below*.

1. How many visits did you have with the mental health professional? ___________ visits
2. Do you know what type of mental health professional(s) you’ve seen? If so, please list their titles (e.g., counsellor, psychologist, psychiatrist) ___________________________________________________________________________
3. How helpful was the visit to the mental health professional? (Please circle)

Extremely unhelpful Extremely helpful

1 2 3 4 5

1. **Actual Help-seeking Questionnaire**: The following list of questions will assess your actual help-seeking experience for your substance use and mental health conditions

| Below is a list of sources or people whom you might seek help or advice from if you were experiencing a personal or emotional problem.  ***Tick*** any of these whom you have gone to for advice or help in the **past 2 weeks** for your substance use or mental health conditions and briefly describe the type of problem you went to them about. | | | |
| --- | --- | --- | --- |
| **S.#** | **Source of Help** | **Yes, briefly describe the type of problem** | **No** |
|  | Intimate partner (e.g. boyfriend/girlfriend/ husband/wife) |  |  |
|  | Friend (not related to you) |  |  |
|  | Parent |  |  |
|  | Other relative / family member |  |  |
|  | Mental health professional (e.g., school counsellor, psychologist, psychiatrist) |  |  |
|  | Religious leader (ex. Du’a, Tselot) |  |  |
|  | Religious places (ex. holy water) |  |  |
|  | Traditional healer |  |  |
|  | General practitioners |  |  |
|  | Nurses or other health professionals |  |  |
|  | Teacher (year advisor, classroom teacher) |  |  |
|  | Someone else not listed above (please describe who this was) ________________ |  |  |
|  | I would not seek help from anyone |  |  |
